# Body Composition, Strength, Mobility, and Posture Associated with Shoulder Pain and Disability in Community-Dwelling Older Adults: A Narrative Review

**DOI:** 10.3390/ijerph23081065

**Published:** 2026-08-17

**Authors:** Yarin Gohar, Ella Been, Leonid Kalichman

**Affiliations:** 1 Recanati School for Community Health Professions, Department of Physical Therapy, Faculty of Health Sciences, Ben-Gurion University of the Negev, Beer-Sheva 84105, Israelbeenella@ono.ac.il (E.B.); 2 Department of Sports Therapy, Faculty of Health Professions, Ono Academic College, Kiryat 55107, Israel

**Keywords:** shoulder pain, shoulder disability, body composition, strength, range of motion, forward head posture, community-dwelling older adults

## Abstract

**Highlights:**

**Public health relevance—How does this work relate to a public health issue?**

- Shoulder pain affects roughly one in five community-dwelling older adults and is a leading contributor to functional decline and loss of independence in an aging global population.
- Current assessment of shoulder pain in this population relies heavily on structural imaging, which correlates poorly with symptoms and disability, contributing to inefficient use of health care resources.

**Public health significance—Why is this work of significance to public health?**

- This review shows that body composition, muscle strength, mobility, and posture are each associated with shoulder pain and disability, identifying modifiable targets for prevention rather than pathology-focused treatment alone; however, the strength of this evidence varies across domains, and evidence for forward head posture in older adults specifically remains limited and largely indirect.

**Public health implications—What are the key implications or messages for practitioners, policy makers and/or researchers in public health?**

- Practitioners and health systems should incorporate brief, low-cost functional screening (strength, range of motion, posture) into the routine assessment of older adults, rather than relying on imaging findings alone.
- Policy makers and researchers should support multicomponent, exercise-based prevention and rehabilitation programs and prioritize longitudinal research that measures these interacting domains concurrently in community-dwelling older adults.

**Abstract:**

Shoulder pain ranks among the leading causes of functional decline in older adults, affecting approximately one in five community-dwelling individuals and contributing to loss of independence and reduced quality of life. Despite its prevalence, shoulder pain in this population is frequently underreported and poorly understood beyond structural pathology, limiting early identification and targeted intervention. This narrative review synthesizes current evidence on the associations between four interrelated physical domains, body composition, grip and shoulder muscle strength, cervical–shoulder range of motion (ROM), and postural alignment, with particular emphasis on forward head posture and shoulder pain and disability in community-dwelling adults aged 60 years and older. A structured search of major electronic databases, including PubMed and Web of Science, was conducted using predefined eligibility criteria and keyword combinations linking shoulder pain and disability with each physical domain. Additional primary studies were identified through manual reference screening of key articles and citation tracking. Evidence was synthesized thematically, highlighting patterns of association, methodological inconsistencies, and contradictions across studies. Findings indicate that higher adiposity, reduced lean mass, shoulder and grip strength deficits, restricted glenohumeral and cervical ROM, and postural deviations are each associated with greater shoulder pain and disability. However, the evidence is not uniformly consistent: associations between Body Mass Index and shoulder ROM are largely absent at moderate adiposity levels, resting postural deviation does not reliably distinguish individuals with shoulder pain from those without, and the causal direction between strength deficits and postural deterioration remains debated. Despite these contradictions, emerging evidence suggests that these domains may interact with one another, and the literature collectively supports an integrated, multifactor assessment framework over single-variable approaches for the evaluation and management of shoulder dysfunction in community-dwelling older adults. Evidence quality was not formally assessed, and evidence for forward head posture specifically in older adults remains limited and largely indirect, drawn mainly from younger populations.

## 1. Introduction

Shoulder pain is one of the most prevalent musculoskeletal complaints worldwide, affecting approximately 1 in 5 older adults and ranking among the leading contributors to functional decline, reduced independence, and diminished quality of life [1,2]. With aging, the cumulative impact of biological changes, including loss of muscle mass, increased adiposity, decreased joint mobility, altered neuromuscular control, and postural deterioration, may create conditions that increase susceptibility to shoulder symptoms. These age-related shifts affect not only the glenohumeral joint but also the shoulder girdle, cervical spine, and thoracic region, collectively influencing shoulder biomechanics and movement efficiency. Importantly, shoulder pain in this population is frequently underreported to healthcare providers, and structural findings such as rotator cuff tears correlate poorly with symptom presence, suggesting that functional and physical capacity factors play a more central role in symptom burden than traditionally recognized [3,4]. Despite extensive research, much of the existing literature examines single risk factors in isolation—rotator cuff pathology, body mass index (BMI), reduced strength, limited ROM, or postural malalignment [3,5,6]. While each factor is individually relevant, this approach overlooks their interplay. Emerging evidence suggests that these domains may interact with one another: excess adiposity and reduced lean mass may exacerbate neuromuscular weakness; strength deficits may impair postural control; and postural deviations may further restrict mobility, potentially worsening pain and disability. Moreover, the existing evidence is not without contradiction: some studies report no significant association between BMI and shoulder ROM, while others find no relationship between resting postural deviation and shoulder pain. However, few studies have investigated these interrelated mechanisms within the same cohort, and, to our knowledge, few have focused specifically on community-dwelling older adults using a comprehensive, multifactor assessment framework. The purpose of this narrative review is therefore to synthesize current evidence on the associations between body composition, grip and shoulder muscle strength, cervical–shoulder ROM, and postural alignment with shoulder pain and functional disability in community-dwelling older adults, and to identify gaps and contradictions in the existing literature that warrant further investigation. This review first examines the evidence for each of the four physical domains in turn, then their interactions with one another, before discussing the clinical and research implications of an integrated, multidomain approach.

## 2. Methods

This article is a narrative review, intended to provide a conceptual, thematically organized synthesis of the literature on multidomain contributors to shoulder pain and disability rather than an exhaustive systematic evidence synthesis. The structured search and eligibility criteria described below were applied to ensure a transparent and reproducible selection process; however, formal systematic review reporting elements, such as a PRISMA-style flow diagram documenting the full screening yield at each stage, were not used, consistent with standard narrative review methodology.

### 2.1. Search Strategy

A structured literature search was conducted across major electronic databases, including PubMed and Web of Science, supplemented by hand-searching of high-yield journals, including the *American Journal of Roentgenology*, the *Journal of Orthopedic and Sports Physical Therapy*, and *Frontiers in Aging*. The search combined controlled vocabulary keywords (MeSH terms) and free-text terms related to shoulder pain and disability, aging, body composition, muscle strength, posture, and mobility. Core search terms included: “Shoulder Pain”, “Shoulder Disability”, “Body Composition”, “Sarcopenia”, “Obesity”, “Grip Strength”, “Shoulder Strength”, “Range of Motion”, “Cervical Mobility”, “Posture”, “Forward Head Posture”, “Craniovertebral Angle”, “Kyphosis”, “Older Adults”, “Community-Dwelling Older Adults”, and “QuickDASH”. Boolean operators (AND, OR) and phrase matching were applied to combine terms across domains (e.g., “body composition AND shoulder pain AND older adults”). Searches were restricted to peer-reviewed articles published in English. The last search update was performed in June 2026. Additional studies were identified through manual screening of reference lists of key included articles and forward citation tracking.

The search was completed on 30 June 2026. This process yielded the 83 sources cited throughout this review, the majority constituting core included evidence and a smaller subset serving as contextual or background citations, as described in Section 2.2.

### 2.2. Inclusion and Exclusion Criteria

Population: Adults aged 60 years or older, with a focus on community-dwelling or independently living older adults. Studies examining younger populations were included when they provided normative data, established measurement validity, or addressed mechanistic relationships relevant to the domains of interest.

Outcomes: Quantitative assessment of shoulder pain intensity and/or functional disability using validated instruments, including but not limited to the Numeric Rating Scale (NRS), Shoulder Pain and Disability Index (SPADI), Visual Analog Scale (VAS), Disabilities of the Arm, Shoulder and Hand (DASH), or QuickDASH.

Exposures: At least one of the four physical domains of interest: body composition metrics (e.g., fat mass, lean mass, BMI, skeletal muscle index), grip or shoulder muscle strength, cervical and/or glenohumeral ROM, or postural parameters including craniovertebral angle (CVA), thoracic kyphosis, or rounded shoulder posture.

Study design: Observational studies (cross-sectional, cohort, or case–control), interventional studies reporting relevant baseline associations, and validation or normative studies of measurement instruments used to assess the defined exposures or outcomes.

Measurement tools: Validated or clinically recognized assessment instruments, including bioelectrical impedance analysis (BIA), dual-energy X-ray absorptiometry (DXA), handheld or isokinetic dynamometry, inclinometer, goniometry, photogrammetry, or validated computer-assisted software.

Studies were excluded if they met any of the following criteria: focused exclusively on surgical populations, competitive athletes, or individuals with acute traumatic injury; involved institutionalized older adults such as nursing home residents; included populations with cognitive impairment likely to bias self-reported pain or disability outcomes; lacked quantitative outcomes directly related to shoulder pain or functional disability; or comprised non-peer-reviewed materials, conference abstracts, or publications in languages other than English. Studies providing normative, biomechanical, or body-composition data without direct shoulder pain or disability outcomes are cited as contextual or background evidence rather than counted among the core evidence base; these are identified as such at the point of citation in the Section 3. The single conference abstract cited in Section 3.6 is likewise presented only as preliminary, hypothesis-generating background evidence pending peer-reviewed confirmation, consistent with its exclusion from the core evidence base.

### 2.3. Data Mapping and Extraction

Data from selected studies were systematically extracted into a structured matrix. For each study, the following variables were recorded: study design, sample characteristics (age, sex, sample size), country of origin, assessment instruments, physical variables investigated (e.g., body composition metrics, strength measures, ROM parameters, postural indices), and primary outcomes related to shoulder pain intensity or functional disability. Where available, quantitative effect estimates including correlation coefficients, odds ratios, regression coefficients, and standardized effect sizes were extracted to enable cross-study comparisons. Associations were organized according to the four predefined physical domains: body composition, muscle strength, mobility, and posture, as well as cross-domain interactions. The synthesis followed a narrative approach, emphasizing patterns of association, methodological consistency, and areas of contradiction or uncertainty across studies. Studies were grouped thematically rather than solely by design, enabling the integration of evidence across disciplines, including biomechanics, gerontology, and physiotherapy.

A summary of representative studies across the four physical domains, including population characteristics, outcome measures, and main associations, is presented in Table 1. Table 1 presents illustrative, not exhaustive, examples of the evidence discussed within each domain; the complete evidence base comprises the 83 sources cited throughout this review, which are distinguished in the text between core included evidence and contextual/background citations, as noted in Section 2.2.

## 3. Results and Synthesis

### 3.1. Shoulder Pain and Disability in Older Adults

Shoulder pain affects approximately 20% of adults aged 65 and over, trailing only back and knee pain, and is more common in women, with approximately 70% attributable to rotator cuff pathology [1,22]. Despite this prevalence, a large proportion of individuals never report symptoms to healthcare providers, leading clinic-based samples to underestimate the true burden [23]. The functional consequences are substantial: compared with pain-free peers, community-dwelling older adults with shoulder pain demonstrate significantly lower American Shoulder and Elbow Surgeons (ASES) scores, reduced shoulder flexion and abduction ROM, and greater role limitations on the 36-Item Short Form Health (SF-36) Survey, with no differences in BMI, comorbidity, or supraspinatus tear prevalence on MRI between groups, suggesting that functional deficits are driven by pain itself rather than structural or systemic factors [7]. Shoulder dysfunction may also contribute to broader mobility limitations through impaired arm swing and reduced capacity for activities of daily living [24], and longitudinal data from 2337 adults followed over four years show that pain tracks are dynamic: 14.6% developed incident pain, recurrent pain was independently predicted by reduced ROM, depressive symptoms, and smoking, and higher grip strength at baseline predicted greater likelihood of pain resolution [8]. Historically, rotator cuff tears were considered the primary driver of shoulder symptoms, but this assumption is increasingly challenged. Full-thickness tears are present in 16.9% of asymptomatic shoulders and reach 50% prevalence in the eighth decade, while large-scale MRI data from 602 adults found no significant difference in full-thickness tear prevalence between symptomatic and asymptomatic shoulders after adjustment [3,4,25]. Together, these findings suggest that shoulder pain in older adults is more closely associated with dysfunctional capacity than with structural pathology, and that clinical assessment should focus on function rather than on imaging alone.

### 3.2. Body Composition and Shoulder Pain and Disability

Body composition refers to the relative proportions of fat mass, lean mass, and bone mass in the body. The gold standard for body composition assessment is DXA, which provides compartment-specific measurements. With aging, skeletal muscle mass declines (sarcopenia) while fat mass increases, particularly visceral and intramuscular adiposity, affecting strength, function, and joint loading [18]. These changes have been linked to greater shoulder pain through three main pathways: mechanical loading from excess adiposity, low-grade systemic inflammation, and impaired muscle quality from reduced lean mass [9,10,11]. Higher body fat mass is associated with greater shoulder pain severity in a threshold-dependent manner. Obese individuals score significantly higher on both VAS and SPADI than normal-weight and overweight groups, with no significant difference between the latter two, and an elevated erythrocyte sedimentation rate implicates systemic inflammation as a likely mechanism [9]. Reduced lean mass contributes through a separate pathway: in a population-based BIA of adults with a mean age of 64.3 years, lower total body muscle mass, lower total body water, lower appendicular muscle mass, were each independently and negatively associated with shoulder pain intensity on a VAS [10], while trunk-specific parameters showed no significance, pointing to peripheral rather than axial muscle reserve as the key factor. In the same study, only the lower total body water ratio remained independently significant after demographic adjustment, suggesting that reduced lean tissue hydration may reflect the most clinically relevant compositional change. The most direct evidence in adults aged ≥65 comes from Han et al. [11], who found shoulder pain prevalence more than twice as high in sarcopenic compared to non-sarcopenic community-dwelling older adults (25.0% vs. 10.7%), with sarcopenia independently associated with supraspinatus tendon thickening and reduced echogenicity across all four rotator cuff tendons, consistent with tendinopathic degeneration rather than structural rupture. Both grip strength and skeletal muscle index correlated positively with tendon echogenicity, supporting a cyclical model in which sarcopenia drives glenohumeral instability and impingement, provoking tendinopathy and pain, which, in turn, promote inactivity and further muscle loss.

In summary, both excess adiposity and reduced lean mass independently contribute to shoulder pain through different pathways. The evidence is not fully consistent. Fat mass did not remain significant after demographic adjustment across all models, and BMI alone is insufficient as a screening tool; however, these findings support body composition assessment as a meaningful component of shoulder pain evaluation in older adults.

### 3.3. Upper Extremity Strength and Shoulder Pain and Disability

Strength deficits are among the most consistent correlates of shoulder pain and dysfunction in older adults, arising from both age-related neuromuscular decline and rotator cuff pathology [5,12,26,27]. Pooled evidence from eight studies confirms that individuals with rotator cuff-related shoulder pain show significantly reduced internal rotation strength compared to asymptomatic peers (Hedges’ g = −0.30). In contrast, external rotation and abduction strength do not reach significance in pooled analysis, suggesting that internal rotation impairment, reflecting subscapularis involvement, is the most reproducible deficit [5]. Within a symptomatic group, however, both abduction (OR = 1.18/kg) and external rotation strength (OR = 1.29/kg) are independently associated with supraspinatus tear presence, though abduction strength in the tear subgroup is more strongly associated with age, sex, and pain severity than with structural factors such as tear size or fatty infiltration [12]. Together, these findings suggest that strength loss in older adults reflects the combined influence of aging, pain, and neuromuscular decline rather than structural pathology alone.

Grip strength extends this picture beyond the shoulder joint. It is independently linked to mortality, disability, sarcopenia, cardiovascular disease, and cognitive decline, and ranks third among global population-attributable risk factors for overall mortality [28,29]. Directly relevant to shoulder function, grip strength is significantly lower in older adults with non-specific shoulder pain compared to pain-free controls (20.78 vs. 24.63 kg, *p* = 0.032), with a minimal clinically important difference of 3.84 kg, and is independently influenced by sex, age, and shoulder pain status, confirming that it reflects overall upper limb function rather than hand strength alone [13]. In summary, both shoulder-specific and grip strength assessments provide clinically meaningful information that imaging alone cannot capture, and both should be interpreted in the context of age, sex, and health status rather than solely based on structural findings.

### 3.4. Cervical–Shoulder Mobility and Shoulder Pain and Disability

Restricted ROM, both glenohumeral and cervical, consistently emerges as a meaningful contributor to shoulder pain and disability in older adults, altering joint mechanics and increasing compensatory muscular activation. Pooled evidence from eight observational studies confirms that rotator cuff-related shoulder pain is characterized by large, multi-planar shoulder ROM deficits, particularly in flexion, external rotation, and internal rotation, with the rotational planes most critical to overhead function showing the greatest impairment [5]. At the individual study level, shoulder abduction and rotation ROMs show the strongest associations with both pain severity and disability, with correlation coefficients ranging from −0.710 to −0.747 against NRS and SPADI scores, and external rotation ROM emerging as the strongest independent contributor to disability [14,30]. Notably, age compounds these deficits, with ROM correlating negatively with age across all planes (ρ = −0.40 to −0.47), highlighting the vulnerability of older adults [14]. In community-dwelling older adults specifically, shoulder ROM shows strong correlations with validated disability measures, including DASH, ASES, and SST scores, and moderately correlates with broader physical performance measures, including gait speed and stair-climb power [24,31]. Importantly, standard ADL scales may miss clinically meaningful shoulder dysfunction that ROM assessment can detect, as nearly 48% of community-dwelling older adults with supraspinatus tears scored normally on functional screens [24]. The cervical spine also correlates with shoulder pain, demonstrates significantly reduced cervical extension ROM compared to pain-free controls, and cervical extension ROM, together with upper trapezius pressure pain threshold, account for 40.2% of the variance in shoulder pain probability [15]. This highlights the need to assess both regions together in clinical evaluation.

In summary, glenohumeral ROM deficits are strongly and consistently associated with shoulder pain and disability, compounded by aging, and reflect broader physical capacity in older adults. Reduced cervical ROM makes an independent contribution, supporting the need to assess both the shoulder and cervical spine together. The causal direction between ROM restriction and pain onset, however, remains unclear from cross-sectional data alone.

### 3.5. Posture and Shoulder Pain and Disability

Postural alignment plays an important role in shoulder biomechanics, as the shoulder girdle, cervical spine, and thoracic spine collectively influence scapular mechanics and shoulder function [16,20,32]. Suboptimal postural alignment alters the resting position of the scapula, disrupts scapulohumeral rhythm, and reduces the subacromial space during arm elevation, increasing load on the rotator cuff and contributing to pain, restricted ROM, and functional disability. Two postural deviations are particularly relevant in older adults: thoracic kyphosis and FHP. Of the two, evidence for thoracic kyphosis is more directly relevant to older adults, whereas evidence for FHP, discussed below, remains largely indirect and drawn from younger or asymptomatic samples. Thoracic kyphosis affects 20-40% of older adults and increases progressively with age, with consequences extending beyond the spine to physical function, disability, and mortality [33]. In community-dwelling older adults, increased kyphosis independently predicts limited shoulder flexion and abduction, a pattern termed “spine–shoulder syndrome” and is associated with greater difficulty reaching overhead and performing daily tasks [16,34]. In younger hyperkyphotic cohorts, kyphosis angle correlates strongly with shoulder pain severity and ROM loss [35], though direct generalizability to older adults is limited.

FHP is defined as anterior displacement of the head relative to the vertical axis, accompanied by upper cervical hyperextension and lower cervical flexion, and is a common postural deviation estimated to affect a large proportion of the patient population [17]. FHP is linked to rounded shoulder posture through muscular imbalances that promote scapular protraction and anterior tilt, disrupting scapulohumeral mechanics [17]. Given that CVA declines 1.6° per decade, older adults face increasing baseline risk [36]. Both deviations may alter shoulder mechanics prior to symptom onset: individuals with FHP and rounded shoulder posture display greater scapular internal rotation, anterior tilting, and reduced serratus anterior activity during arm elevation, independent of pain [32]. The functional consequences of FHP are well documented in younger populations, specifically where it co-occurs with higher DASH and NDI scores, with CVA explaining 43% of the variance in upper-limb disability [17]. Direct evidence in adults aged 60 and over, however, remains limited. An important contradiction between the two deviations comes from Barrett et al. [6], whose systematic review of 2794 participants found strong evidence that erect posture immediately improves shoulder ROM in both symptomatic and asymptomatic individuals, yet only moderate evidence of no significant difference in resting kyphosis between those with and without shoulder pain. This points to dynamic postural capacity, the ability to achieve thoracic extension during movement, as more clinically relevant than static angle alone.

In summary, thoracic kyphosis and FHP alter scapular mechanics, restrict shoulder and cervical ROM, and are associated with greater upper limb disability, yet static resting posture does not reliably distinguish those without shoulder pain. Postural assessment should focus on movement quality during arm elevation rather than on resting angle alone, and postural correction should be considered a component of a broader approach rather than a standalone intervention.

### 3.6. Interaction Between Domains

**Figure 1:**
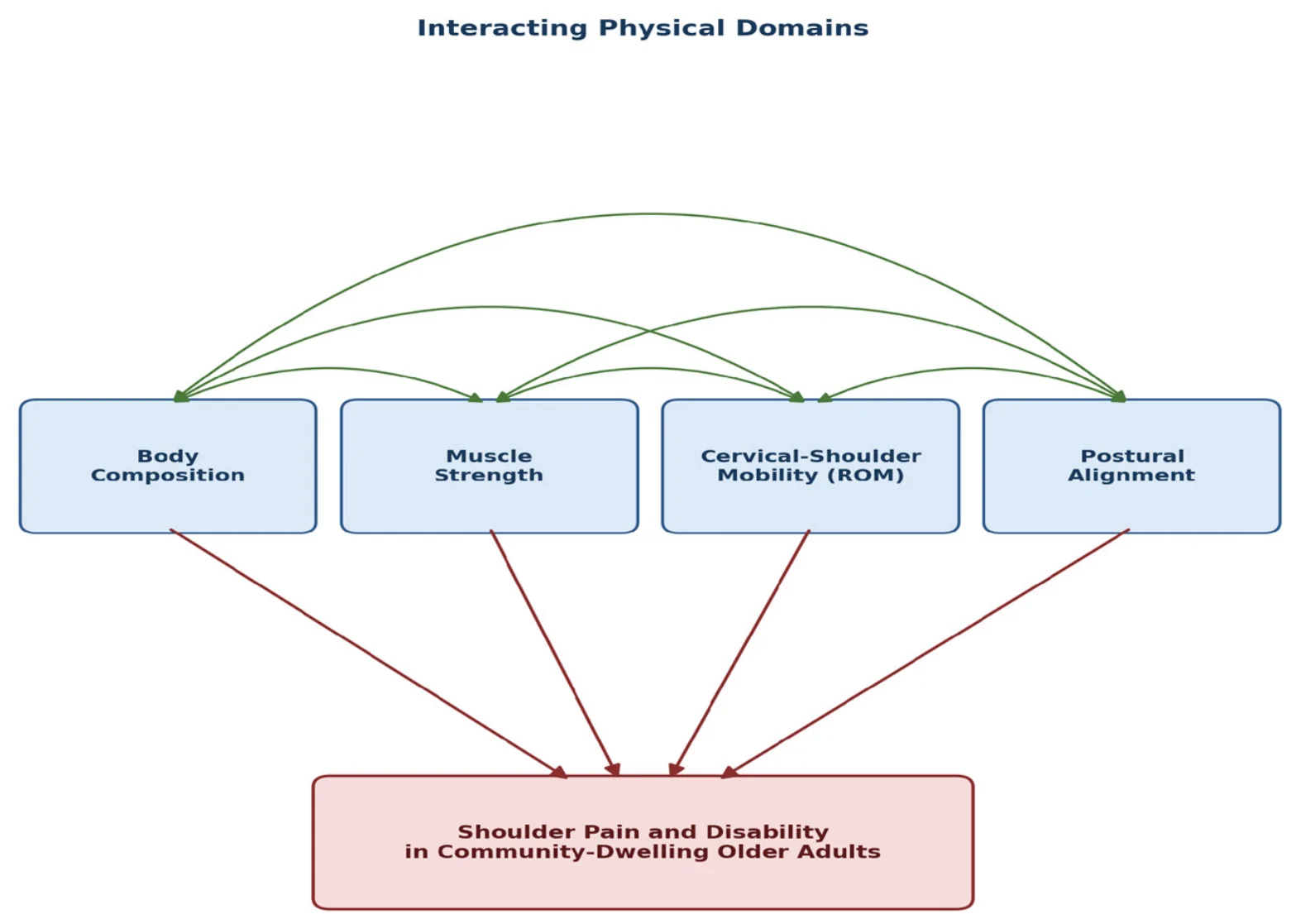
Conceptual Model of Interacting Physical Domains Associated with Shoulder Pain and Disability in Community-Dwelling Older Adults. Note: Green double-headed arrows represent bidirectional interactions between physical domains (see Section 3.6). Red arrows indicate the association between each domain and shoulder pain and disability. Arrow direction does not imply established causality; most underlying evidence is cross-sectional.

Figure 1 summarizes these interactions as a hypothesis-generating conceptual model, illustrating how body composition, muscle strength, cervical–shoulder mobility, and postural alignment may relate to one another and to shoulder pain and disability; the underlying evidence for these pathways is drawn largely from separate populations and study designs rather than from integrated testing in the same cohort.

Body Composition and Upper Extremity Strength: Lean mass is the primary determinant of grip and upper extremity strength throughout lifespan. Evidence from children to older adults consistently shows that fat-free mass and skeletal muscle mass are the strongest predictors of grip strength, while percentage body fat exerts an independent, negative, but smaller effect [37,38,39,40]. In older adults, lean body mass is a strong independent predictor of grip strength, with multivariable models incorporating lean body mass and other relevant factors explaining approximately 47–54% of the variance in hand-grip strength), whereas BMI alone is not a significant predictor [18,41]. 

For shoulder-specific strength, the picture is less consistent. Obesity appears to increase absolute shoulder flexion strength through mechanical loading, yet simultaneously impairs muscular endurance and functional capacity, with fat infiltration independently associated with reduced muscle strength [42,43,44]. However, when physical activity is controlled, no significant association between BMI and shoulder strength emerges [45], suggesting that fitness level moderates this relationship.

Body Composition and Mobility: The relationship between body composition and upper-extremity and cervical ROM is inconsistent and context-dependent. Where significant associations exist, they tend to be selective rather than globally restricted to specific movement directions in which adipose tissue mechanically obstructs motion, and most apparent at higher BMI levels [46,47]. Obesity has been shown to reduce shoulder extension and adduction but not flexion, abduction, or rotation, reflecting the anatomical distribution of excess fat relative to movement planes [46]. A dose–response pattern has been observed, with clinically meaningful ROM reductions emerging primarily at obese and morbidly obese BMI levels rather than at overweight levels [47,48,49]. In contrast, several studies found no significant relationship between BMI and shoulder or cervical ROM, including in patients with chronic shoulder pain [50], young overweight adults [51], and across cervical movement directions in neck-healthy adults [52]. These contradictions likely reflect differences in body composition measurement, the specific joints and movement directions examined, the presence or absence of pain, and population characteristics.

Body Composition and Posture: Body composition influences upper body posture through two distinct pathways. Excess adiposity, particularly in abdominal and visceral regions, promotes anterior displacement of the center of gravity, driving compensatory increases in thoracic kyphosis and FHP across multiple populations and measurement methods [53,54,55]. This relationship extends to FHP specifically, with BMI independently and negatively correlated with CVA in both women and men, and obese individuals showing 1.34 times greater risk of FHP than non-obese counterparts [56,57]. Visceral fat may be particularly relevant, with one preliminary study reporting that it independently predicted CVA beyond BMI alone, potentially mediated through fascial line transmission, though this finding is based on a conference abstract and requires peer-reviewed confirmation [58]. Importantly, reduced lean mass represents a second independent pathway. In community-dwelling adults aged 65+, lower trunk lean mass was significantly associated with hyperkyphosis across multiple measurement methods, whereas fat mass showed no consistent association [59]. This suggests that preserving trunk muscle mass may be as important as managing adiposity for maintaining spinal alignment in older adults.

Upper Extremity Strength and Mobility: Shoulder strength and ROM decline in parallel with aging, suggesting a shared physiological basis attributable to sarcopenia, capsular stiffening, and declining neuromuscular coordination [26,27]. However, their relationship is not linear. Excessive or imbalanced strength development may paradoxically restrict ROM. In an extreme example illustrating this tradeoff outside the review’s target population, bodybuilders demonstrate significantly greater strength yet reduced internal rotation ROM compared to non-bodybuilders [60]; in the context of pathology more relevant to the target population, strength and ROM deficits are frequently observed to co-occur [61]. In the cervical region, the strength–ROM relationship is less predictable. Despite the largest cervical biomechanics cohort to date finding no difference in cervical ROM or torque between individuals with and without chronic neck pain [62], individuals with shoulder pain demonstrate significantly reduced cervical ROM and neck muscle endurance across all planes compared to pain-free controls [63]. This suggests that cervical and shoulder dysfunction are linked, and that both regions should be assessed together in clinical evaluation.

Upper Extremity Strength and Posture: The strength–posture relationship is bidirectional. FHP and rounded shoulder posture alter scapular muscle recruitment, increasing upper trapezius activity and reducing serratus anterior activity during shoulder elevation. Both voluntary and therapist-corrected postural corrections can normalize this pattern [64,65]. Notably, rounded shoulder posture and FHP appear to be largely independent conditions that differentially influence muscle recruitment, with CVA correlating with neck disability but not with rounded shoulder posture [66,67]. FHP is also associated with reduced cervical flexor and extensor strength, plausibly through altered length–tension relationships, and with reduced cervical muscle endurance relative to maximal isometric strength [68].

In the opposite direction, reduced muscle strength contributes to postural deterioration. Back extensor and periscapular strength are each independently associated with kyphosis severity [69,70,71], and low grip strength prospectively predicts kyphosis progression over four years, suggesting it is a modifiable risk factor rather than simply a consequence [19]. Cross-sectional associations between kyphosis angle and grip strength are consistent across large cohorts [72,73,74], though the relationship between head–neck position and grip strength is inconsistent in young asymptomatic populations [75,76]. Importantly, this cycle of weakness and postural deterioration is amenable to exercise intervention [77].

Mobility and Posture: Thoracic kyphosis, FHP, and rounded shoulder posture are each consistently associated with reduced shoulder and cervical ROM across experimental, cross-sectional, and systematic review designs. Experimental studies demonstrate a clear dose–response relationship between increasing thoracic kyphosis and reduced shoulder flexion, abduction, and external rotation, with even a modest 7° increase in kyphosis producing clinically meaningful ROM reductions, and these effects occur independently of pain [20,78,79]. A systematic review of 10 studies found strong evidence that an erect posture consistently improves shoulder ROM in both symptomatic and asymptomatic individuals, whereas the resting kyphosis angle alone does not distinguish those with shoulder pain from those without [6]. For the cervical spine, smaller CVA is consistently associated with reduced ROM across all planes, and FHP has been shown to mediate the relationship between thoracic kyphosis and cervical mobility through a sequential pathway in which kyphosis drives FHP, which in turn restricts cervical ROM [21,80,81]. These associations are also clinically relevant in older adults with habitual postural deviations [82] and extend to glenohumeral ROM in younger adults with FHP [83].

## 4. Discussion

### 4.1. Interpretation of Findings

The reviewed evidence consistently points to shoulder pain and disability in older adults as a multidomain problem rather than a single-variable one. Body composition, muscle strength, cervical–shoulder mobility, and postural alignment are each associated with shoulder dysfunction and appear to interact with one another: lean mass is strongly associated with grip and shoulder strength [18,41]; kyphosis and FHP are associated with restricted ROM through shared biomechanical pathways [20,21]; and grip weakness prospectively predicts postural deterioration [19]. These interactions suggest that single-domain assessment underestimates the true clinical picture. A key contradiction runs through the evidence: structural findings, rotator cuff tears, and resting kyphosis angle do not reliably distinguish those with from those without shoulder pain [3,4,6], while functional measures such as strength, ROM, and dynamic postural capacity distinguish these groups more consistently. This may support a shift toward function-based evaluation as a complement to, rather than a replacement for, imaging-centric assessment in this population. Finally, several associations between BMI and ROM, and between FHP and grip strength, are only meaningful at higher adiposity levels or in older adults with hyperkyphosis [47,74], suggesting that physical impairments reach clinical significance thresholds only in the context of aging or concurrent pathology. Longitudinal studies focused specifically on community-dwelling older adults are needed to establish causal direction.

### 4.2. Implications for Clinicians

We propose that clinicians consider incorporating brief functional screening into the assessment of older adults presenting with shoulder pain, as a complement to structural imaging rather than a replacement for it. A pragmatic assessment battery might include grip and shoulder strength testing (handheld dynamometry), active glenohumeral and cervical range of motion (goniometry), a simple postural screen (e.g., craniovertebral angle or a flexicurve-based kyphosis index), and a validated self-report measure such as the QuickDASH or SPADI. This proposed approach follows from the associations identified in this review and has not itself been tested as an integrated assessment protocol. Identifying deficits across these domains, rather than relying solely on imaging findings, may help clinicians better target rehabilitation strategies such as strength training, postural correction, and combined shoulder–cervical mobility work.

### 4.3. Implications for Researchers

For research, these findings highlight the need for longitudinal cohort studies that concurrently measure body composition, strength, mobility, and posture within the same community-dwelling older adults, enabling mediation and cross-lagged analyses to help clarify temporal and causal relationships. Multidomain, model-based approaches (e.g., structural equation modeling) could help quantify the relative and interactive contributions of each domain to shoulder pain and disability, and may help resolve some of the contradictions identified in this review.

### 4.4. Relevance of Evidence from Younger Populations

A substantial portion of the evidence synthesized in this review, particularly for forward head posture, rounded shoulder posture, and some body composition–mobility associations, originates from younger or mixed-age samples rather than from community-dwelling older adults specifically. These studies were included when they established measurement validity, described mechanistic relationships plausibly relevant across the lifespan, or when direct evidence in older populations was unavailable. However, aging introduces physiological changes, including sarcopenia, degenerative joint and capsular changes, and altered neuromuscular control, that may modify these relationships in ways not captured by younger cohorts. Findings extrapolated from younger populations should therefore be interpreted as hypothesis-generating rather than directly generalizable to community-dwelling older adults; this limitation is flagged at the relevant points throughout the review (e.g., FHP–disability associations). Future studies conducted specifically in older cohorts are needed to confirm whether associations identified in younger populations hold across the aging process.

### 4.5. Strengths and Limitations of Literature

A major strength of the existing body of work is the consistency with which individual physical variables: fat mass, muscle strength, ROM, and postural alignment are linked to shoulder dysfunction across diverse methodologies, populations, and geographical settings. Many studies employ validated clinical tools, such as DXA, BIA, dynamometry, and photogrammetry, thereby enhancing the reliability and comparability of observed associations. Additionally, recent research increasingly considers proximal regions such as the cervical spine, reflecting greater awareness of the connection between the neck and shoulder in clinical assessment.

However, the evidence has several important limitations:

- This review did not apply a formal risk-of-bias or quality-appraisal tool; cross-sectional, longitudinal, experimental, and indirect evidence from non-target populations were not formally weighted, which may overstate the certainty of associations drawn from lower-quality or indirect study designs.
- Most included studies used cross-sectional designs, which prevent conclusions about causality and limit the ability to determine whether physical impairments precede or follow the onset of shoulder pain.
- Sample sizes were frequently small and heterogeneous, reducing statistical power and complicating cross-study comparison.
- Measurement protocols varied widely, particularly for strength and postural assessment, resulting in inconsistent effect sizes and making cross-study comparisons difficult.
- The age ranges of many included studies were broad, and relatively few focused exclusively on adults aged 60 and over, limiting direct generalizability to the community-dwelling older adult population of primary interest.
- Most studies examined physical domains in isolation rather than concurrently, restricting insight into how body composition, strength, mobility, and posture interact within the same individual across the aging process.
- Some important controversies remain unresolved, notably whether FHP is a true biomechanical driver of shoulder symptoms or a compensatory postural adaptation to pain and dysfunction, highlighting the need for more mechanistic and longitudinal research designs.

Overall, the fragmented, largely single-domain evidence base, evaluated without formal quality weighting, underscores the need for studies that assess these variables together in community-dwelling older adults.

## 5. Conclusions

Shoulder pain and disability in community-dwelling older adults are a multifactorial problem that cannot be understood through a single-variable assessment. Body composition, muscle strength, cervical–shoulder ROM, and postural alignment are each associated with shoulder pain and disability, and structural pathology such as rotator cuff tears shows poor correspondence with symptom presence across all four domains. Together, this evidence supports a function-based assessment as a complement to imaging, rather than imaging alone, when evaluating shoulder complaints in this population.

Important gaps remain. Most of the underlying evidence is cross-sectional and was not formally appraised for quality or risk of bias, so the certainty and causal direction of these associations remain unclear. We were unable to identify studies that concurrently evaluated all four domains within the same group of older adults, and evidence linking FHP specifically to shoulder pain in adults aged 60 and over remains largely indirect, drawn mainly from younger populations. Future longitudinal studies conducted specifically in community-dwelling older adults, examining all four domains together over time and applying formal quality appraisal, would help clarify the temporal relationships between these physical impairments and shoulder pain, and could support earlier, more targeted prevention in this population.

## Figures and Tables

**Table 1:** Representative Studies Summarizing Associations Between Physical Domains and Shoulder Pain/Disability.

Domain	Study	Population	Outcome Measure	Main Association
Overview	Patel et al. [1]	US community-dwelling adults ≥65 years (NHATS)	Self-reported pain prevalence survey	Shoulder pain is among the most prevalent pain conditions in older adults, after back and knee pain
Overview	Davis et al. [7]	Community-dwelling adults >60 years	ASES, SF-36; MRI tear status	Shoulder pain associated with lower QoL/function independent of BMI, comorbidity, or MRI tear status
Overview	Gill et al. [8]	2337 community-dwelling adults, 4 years follow-up	Self-reported pain incidence/recurrence; grip dynamometry	Reduced ROM, depressive symptoms, and smoking predicted incident/recurrent pain; higher grip strength predicted resolution
Overview	Ibounig et al. [4]	602 Finnish adults, 41–76 years	3-Tesla MRI; symptom questionnaire	Rotator cuff abnormalities are near-universal (99%) and poorly correlated with symptom status
Body composition	Özkuk & Ateş [9]	Adults with chronic shoulder pain	VAS, SPADI; BMI category	Obesity is associated with higher VAS/SPADI scores vs. normal-weight and overweight groups
Body composition	Iizuka et al. [10]	Japanese general population, mean age 64.3 years	VAS; bioelectrical impedance analysis (BIA)	Lower total body muscle mass and body water are independently associated with greater shoulder pain
Body composition	Han et al. [11]	Community-dwelling elders ≥65 years	Ultrasound (tendon echogenicity); pain prevalence	Sarcopenia is associated with >2× higher shoulder pain prevalence and rotator cuff tendon changes
Strength	Manoso-Hernando et al. [5]	8 pooled studies, rotator cuff-related shoulder pain	Isokinetic/handheld dynamometry (strength)	Internal rotation strength deficit is the most reproducible finding (Hedges’ g = −0.30)
Strength	Miller et al. [12]	Symptomatic shoulder pain patients	Dynamometry; MRI tear presence	Abduction and external rotation strength are independently associated with the presence of a supraspinatus tear
Strength	Calvo-Lobo et al. [13]	Older adults with/without shoulder pain	Hand-grip dynamometry	Grip strength was significantly lower in the shoulder pain group (20.78 vs. 24.63 kg)
Mobility	Anwer et al. [14]	Patients with shoulder dysfunction	Goniometry (ROM); NRS, SPADI	Shoulder abduction/rotation ROM strongly correlated with pain severity and disability (r = −0.71 to −0.75)
Mobility	Rebelatto et al. [15]	Individuals with/without shoulder pain	Goniometry (cervical ROM); pressure pain threshold	Cervical extension ROM plus upper trapezius pressure pain threshold explain 40.2% of variance in shoulder pain
Posture	Barrett et al. [6]	2794 participants (systematic review)	Photogrammetry/inclinometer (kyphosis); ROM	Erect posture consistently improves shoulder ROM; resting kyphosis angle does not distinguish pain status
Posture	Imagama et al. [16]	Middle-aged and elderly adults	Radiographic kyphosis angle; shoulder ROM	Increased kyphosis independently predicts limited shoulder flexion/abduction (“spine–shoulder syndrome”)
Posture	Kim & Lee [17]	Adults with forward head posture	Craniovertebral angle (photogrammetry); DASH	Craniovertebral angle explains 43% of the variance in upper limb disability (DASH)
Interaction between domains	Charlton et al. [18]	Adults aged 55–96 years	BIA (lean mass); hand-grip dynamometry	In older adults, lean body mass is a strong independent predictor of grip strength, with multivariable models incorporating lean body mass and other relevant factors explaining approximately 47–54% of the variance in hand-grip strength
Interaction between domains	Sugai et al. [19]	Elderly Japanese, 4 years follow-up (Kurabuchi Cohort)	Grip dynamometry; radiographic kyphosis angle	Low grip strength prospectively predicts kyphosis progression
Interaction between domains	Kebaetse et al. [20]	Healthy adults (experimental)	3D kinematics; ROM; dynamometry	Thoracic posture alters shoulder ROM, strength, and 3D scapular kinematics
Interaction between domains	Quek et al. [21]	Older adults	Craniovertebral angle, kyphosis angle, cervical ROM	Sequential pathway: thoracic kyphosis → forward head posture → restricted cervical ROM

Note. The table presents representative, not exhaustive, studies for each domain discussed in the narrative synthesis. BIA = bioelectrical impedance analysis; DASH = Disabilities of the Arm, Shoulder, and Hand; QoL = quality of life; ROM = range of motion; SPADI = Shoulder Pain and Disability Index; VAS = Visual Analog Scale.

## Data Availability

No new data were generated or analyzed for this narrative review. All data discussed are available in the cited primary sources.
